# Supplementary material for: Development and Characterization of Recombinase-Based Isothermal Amplification Assays (RPA/RAA) for the Rapid Detection of Monkeypox Virus
Source: Viruses. 2022 Sep 23;14(10):2112. doi: 10.3390/v14102112 (PMC9611073; doi:10.3390/v14102112)
Supplement: Supplementary file 1 [file viruses-14-02112-s001.zip › viruses-1891652-supplementary.pdf]

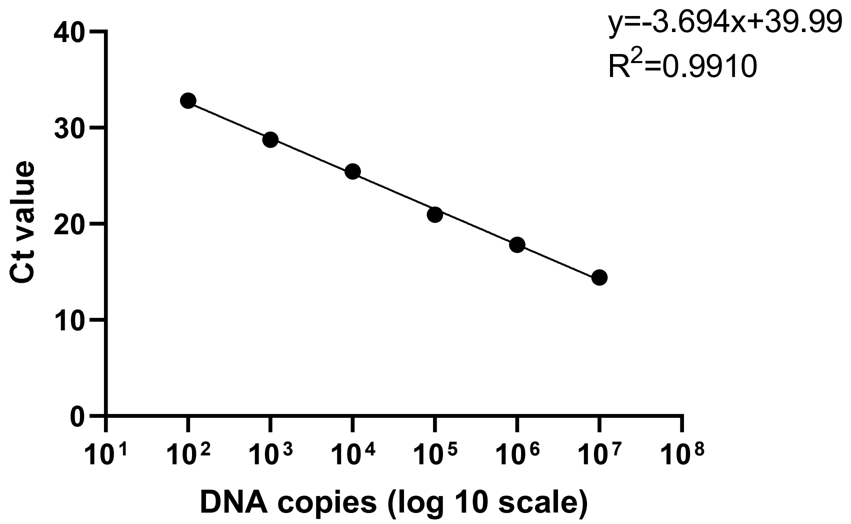

Figure S1. The standard curve of the real-time PCR. Serial 10-fold dilutions of MPXV DNA plasmids (10^7-10^0 copies) were detected by real-time PCR. The standard curve was calculated from the Ct value of 10^7 to 10^2 concentration of MPXV DNA plasmids.
